# Treatment of In-Growing Nail

**Published:** 1869-04

**Authors:** 


TREATMENT OF IN-GROWING NAIL.—By DR. BABB. (*Boston Journal*, July 9; and *Medical Times and Gazette*, August 29.)

Dr. Babb says that for several years he has been in the habit of employing in these cases, with uniform success, a saturated solution of persulphate of iron. A bit of cotton wool saturated with this is insinuated between the fungous flesh and the nail, letting the cotton turn back over the flesh on the outside also. Success depends much on the thoroughness with which the bit of cotton is pushed down to the bottom or edge of the ingrowing nail.

